# “Don’t You Love Me?” Abusers’ use of shame-to-guilt to coercively control 2SLGBTQQIA+ individuals and rural women experiencing intimate partner violence

**DOI:** 10.1177/17455057251335361

**Published:** 2025-04-30

**Authors:** Stefan Kurbatfinski, Nicole Letourneau, Jason Novick, Susanne Marshall, Keira Griggs, Dawn McBride, Kendra Nixon

**Affiliations:** 1 Owerko Centre, Alberta Children’s Hospital Research Institute, University of Calgary, Calgary, AB, Canada; 2 Department of Community Health Sciences, Cumming School of Medicine, University of Calgary, Calgary, AB, Canada; 3 Department of Pediatrics, Faculty of Nursing and Cumming School of Medicine, University of Calgary, Calgary, AB, Canada; 4 Department of Psychiatry, Faculty of Nursing and Cumming School of Medicine, University of Calgary, Calgary, AB, Canada; 5 Department of Community Health Sciences, Faculty of Nursing and Cumming School of Medicine, University of Calgary, Calgary, AB, Canada; 6 Faculty of Education, University of Lethbridge, Lehtbridge, AB, Canada; 7 Faculty of Social Work, University of Manitoba, Winnipeg, MB, Canada

**Keywords:** intimate partner violence, emotional abuse, 2SLGBTQQIA+, rural women, intersectionality, shame-to-guilt, qualitative study

## Abstract

**Background:**

Abusers’ use of manipulative behaviors to trigger feelings of shame-to-guilt (a process through which abusers shame their partners to incur feelings of guilt) among their 2SLGBTQQIA+ and rural women intimate partners is a type of emotional abuse used to coercively control their partners.

**Objective:**

This study investigated the different tactics that abusers use to shame-to-guilt their partners who identify as 2SLGBTQQIA+ and/or reside in rural areas.

**Design:**

A qualitative design was used to conduct this study.

**Methods:**

We used data from two larger studies to undertake thematic analysis using semi-structured interviews with Albertan 2SLGBTQQ+ (*n* = 18; no participants identified as intersex or asexual) and rural women (*n* = 11) who experienced shame-to-guilt behaviors along with service providers who worked with these groups (*n* = 24).

**Results:**

Seven themes were identified based on participants’ experiences, including shaming identity in relation to gender and sexual orientation (manifesting differentially between 2SLGBTQQ+ and rural women participants), emotional and sexual manipulation, threats of death by suicide (predominating among 2SLGBTQQ+ individuals), apologies and vacuous promises as components of the cycle of abuse, using one’s parenting and children’s well-being to manipulate partners, the use of health conditions and faking illness, and the use of religion or faith to reinforce gender standards.

**Conclusion:**

For 2SLGBTQQIA+ and rural women groups, situating shame-to-guilt behaviors within the cycle of abuse is important information that has not been explored extensively in the intimate partner violence literature. For individuals self-identifying as 2SLGBTQQIA+ or women living rurally, the means through which they are shamed-to-guilt intersects with their unique identities and positionality. Therefore, recommendations are presented to help these groups rebuild their identities when shame-to-guilt behaviors were experienced as part of the abusive dynamic.

## Plain language summary

How abusive partners use shame and guilt in abusive relationships: Experiences from 2SLGBTQQ+ and women living rurally along with those who provide formal support

Manipulation can be used by abusive partners to make people feel ashamed and guilty. By doing this, people who feel guilty or ashamed end up feeling bad and sorry for their partners, allowing for the partner to be even more abusive and feel as if they are not to blame for their relationship. Using interviews, we looked at how abusive partners used shaming and guilting behaviors to control their partners in 2SLGBTQQIA+ (18 people) and rural women (11 people) groups. We also looked at how those providing support (24 people) to these groups saw shame and guilt happen with their clients. We took data from two larger reports. Interviews happened over Zoom or telephone. Many findings were found, but seven main things arose: (1) shaming gender or sexual identity, (2) emotional or sexual manipulation, (3) threats of death by suicide, (4) the cycle of abuse and trauma-bonding, (5) targeting parenting skills, (6) faking health conditions, and (7) using religious values to guilt people. It is important to tell people what shame and guilt can look like in abusive relationships so people can identify these behaviors by themselves and seek support when they are ready. We provide some important recommendations and ideas for policy changes.

## Introduction

Emotional abuse constitutes a form of intimate partner violence (IPV) that is non-physical in nature, aiming to control, intimidate, or hurt an individual.^1^ Of those, manipulative behaviors (e.g., gaslighting) that aim to produce feelings of shame and guilt among an intimate partner are critical to highlight as they are injurious, yet subtle in nature, enabling abusers to coercively control their partners.^2,3^ Abused individuals who are made to feel ashamed or guilty may be less likely to disclose their experiences of violence, leave the abusive relationship, or prioritize their personal health, ultimately leading to ongoing connection with their abusive partner and poorer quality of life.^2,4^ Abusers will often employ shame (a feeling inflicted by another person) to guilt (a feeling developed by a person) their partner(s),^2,3^ a process which this teams refers to and coins as “shame-to-guilt.” The shame-to-guilt process is also intrinsically linked with the cycle of abuse which often begins with the build-up of tension until reaching a climactic violent episode that threatens the safety and well-being of the individual being abused.^5^ When the individual experiencing IPV is motivated to leave the relationship, the abusive partner will employ manipulatively apologetic and shame-to-guilt behaviors to maintain the relationship.^5^ If successful, abusers typically engage in more severe behaviors with increased frequency with every cyclic iteration.^5^

Diverse tactics can be employed by abusers to manipulatively shame-to-guilt their partners, strategically utilizing contextual factors unique to their relationship (e.g., social, environmental, financial, health-related).^6^ For example, some abusive partners can develop deliberately strong relationships with their partner’s social networks,^7^ inducing these social networks to become more loyal to the abusive individual and to knowingly or unknowingly shame-to-guilt the individual experiencing IPV into remaining in the relationship.^8^ Mental health conditions can be leveraged by abusers to shame-to-guilt their partners into feeling like they are responsible for the abuse,^9^ preventing them from leaving the abusive relationship.^10^ Similarly, religion can be used by abusers to shame-to-guilt their partners into remaining in the abusive relationship due to entrenched beliefs about gender, commitment, and family responsibilities.^11,12^ These are only few examples of shame-to-guilt behaviors that deliberately target sensitivities and vulnerabilities of those experiencing IPV.

### IPV intersects with different identity groups

There is substantial evidence that IPV intersects uniquely with different identities and social determinants of health due to intrinsic links with social forces (e.g., heteronormativity, geographic remoteness).^13^ Representing a myriad of identities, Two-Spirit, lesbian, gay, bisexual, trans, queer, questioning, intersex, asexual, and other sexual and gender minorities (2SLGBTQQIA+) individuals face increased vulnerability to poor health outcomes compared to their heterosexual counterparts due to discrimination and inequitable access to healthcare services and resources (e.g., promotion of conversion therapy).^14^ Utilization of healthcare services can also bring about other challenges, such as 2SLGBTQQIA+ individuals having to assume the role of educating their healthcare providers while also trying to maintain a positive image of the community (e.g., downplaying the gravity of violence they experienced).^14,15^ The diverse gender identities and sexual orientations within the 2SLGBTQQIA+ community strongly suggests variability in individuals’ experiences of IPV based on their gender and sexuality. For example, in one study, bisexual individuals reported poorer mental health compared to lesbian or gay individuals,^14^ a risk factor for both experiencing and engaging in IPV^1^; this could potentially be explained by the concomitant experiences of exclusion within the 2SLGBTQQIA+ community and biphobic responses that bisexual individuals must navigate.^16^ When examining violence more broadly, older lesbian and bisexual adult women also reported experiencing greater levels of violence than other sexual and gender minority groups and from more than one perpetrator.^17^ Relative to their cisgendered respondents, trans and gender nonconforming individuals were also more likely to experience suicidal thoughts and more likely to attempt death by suicide.^18^ A lack of understanding of how shame-to-guilt behaviors manifest uniquely among 2SLGBTQQIA+ individuals reveals the need for their exploration to help redefine IPV understandings.

Women residing rurally are at an increased risk of experiencing coercively controlling IPV due to geographic isolation.^19^ They may hesitate to access healthcare services due to fear of their ex-partner retaliating, a lack of confidentiality, and of the abuser being in a position of power,^20^ among other factors. Studies completed on rural versus urban IPV rates suggest that rates of experiencing IPV are similar across rural and (sub)urban areas, but that rural individuals experiencing IPV are at an increased risk of worsened health trajectories due to a lack of accessibility to and diversity of care provision.^21^ The geographic disposition of rural living likely diversifies abusers’ capacity to engage in shame-to-guilt behaviors, using the lack of privacy, maintenance of patriarchal gender norms, and lack of services and resources to their advantage.^20^ This emphasizes the need to also examine IPV specifically within the context of rural women groups.

### Purpose of the study

In the field of IPV research, less emphasis is placed on the various tactics that abusers may use to shame-to-guilt their partners, which often precedes other forms of IPV such as physical or sexual abuse and can incur grave impacts on physical and mental health.^4,5^ Therefore, the purposes of this qualitative study were to assess perspectives of isolated individuals experiencing IPV (i.e., 2SLGBTQQIA+ and rural women) and relevant service providers to: (1) identify different tactics that abusers may use to shame-to-guilt their partners during and following the end of the intimate partner relationship and (2) describe the intended goal of using the identified shame-to-guilt behaviors. Identifying these various forms of shame-to-guilt behaviors can help those experiencing IPV to better relate with their experiences of shame-to-guilt and to provide a better understanding of potential behaviors that could be expected to occur within 2SLGBTQQIA+ and rural women groups.

## Methods

### Methodology

Data for this article arose from two concurrent qualitative studies conducted by the Research and Education for Solutions to Violence and Abuse (RESOLVE) Network^22^ which focused on the experiences of IPV for 2SLGBTQQIA+ individuals and rural women living in the three prairie provinces of Canada (i.e., Alberta, Saskatchewan, Manitoba).^23,24^ 2SLGBTQQIA+ and rural women individuals were studied since Alberta, the setting of this study, is considered a more conservative province with high-rates of gender-related homicide in the province,^25^ particularly affecting these two groups. Participants’ perspectives of their lived experiences with IPV were examined, enabling them to describe their stories in their own words. The theory of intersectionality also influenced this study’s design.^26^ This theory posits that individuals hold multiple intersecting positionalities and experience different oppressions, impacting their personal experiences, particularly as it relates to privilege and systemic oppression.^26^ How participants’ identities impacted their experience with IPV and help-seeking provides paramount context in interpretations.^26^ We followed the Consolidated Criteria for Reporting Qualitative Research (COREQ) guidelines when reporting this research (Supplemental Material Table A).^27^ Ethical approval for both studies was obtained from the University of Calgary Conjoint Health Research Ethics Board (REB20-1461_REN3, REB20-1241_REN6).

We used the term “intimate partner violence” to encompass all forms of violence and abuse, regardless of severity or type. This includes patterns of violence and abuse such as physical, emotional, psychological, verbal, identity, religious, legal, sexual, financial, and spiritual as well as aspects of coercive control and stalking. These acts may range from periodic to long-term and minor to severe. Furthermore, an intimate partner was characterized as someone with whom one has had an intimate experience (whether short-term or long-term). This may or may not include aspects of a physical, emotional, psychological, sexual, or financial relationship between the parties. We also avoided using terms such as victim, survivor, or perpetrator, as individuals with lived experience have described stigma and blame associated with the use of those terms.

### Participant eligibility and data collection

Eligibility requirements for self-identifying 2SLGBTQQIA+ individuals and rural women are provided (Table 1). As no intersex and asexual individuals entered the study, we used the acronym 2SLGBTQQ+ moving forward. Data were collected from individuals experiencing IPV through individual, telephone-based, semi-structured interviews. Focus groups were not offered to individuals experiencing IPV as IPV is a sensitive topic and anonymity, safety, and comfort were prioritized. Data were collected from service providers providing relevant services through both individual, semi-structured telephone interviews and focus groups via Zoom. Service providers were permitted to choose if they preferred an individual interview or a focus group. Among service providers providing support to rural women, seven chose to participate in individual interviews while nine were divided into two focus groups. All of the service providers supporting 2SLGBTQQ+ individuals chose to complete individual interviews.

**Table 1.** Eligibility criteria for participants.

Rural women	2SLGBTQQ+ individuals
• Residing in the province of Alberta during their experiences with IPV • Resided in an area with a population of less than 20,000 individuals • 18 years of age or older • Experienced IPV within the previous 10 years • No longer living with their abusive partner • Identified as a woman	• Residing in the province of Alberta during their experiences with IPV • Experienced IPV within the previous 10 years • 18 years of age or older • No longer living with their abusive partner • Self-identified as a 2SLGBTQQ+ individual
Service providers working with rural women	Service providers working with 2SLGBTQQ+ individuals
• Working for an agency or organization serving women residing in rural areas who are experiencing IPV • Able to participate in focus group or individual interviews via video conference and answer questions pertaining to the nature and context of IPV experienced by women in rural communities	• Working for an agency or organization serving 2SLGBTQQ+ individuals experiencing IPV • Able to participate in individual interviews via phone or video conference and answer questions pertaining to the nature and context of IPV in 2SLGBTQQ+ communities

IPV: intimate partner violence.

Though we did not pilot-test the interview guides, a survey which was conducted prior helped to inform the development of the guides. Similar questions were asked to both 2SLGBTQQ+ and women living rurally, focusing on demographic information, their experiences of IPV and with help-seeking, and recommendations to improve how individuals experiencing IPV are supported; however, some differences existed in relation to living rurally or in identifying as a sexual or gender minority (Supplemental Material Interview Guides). For example, 2SLGBTQQ+ individuals were not asked about children for two reasons: (1) survey data collected prior to the interviews suggested parenthood status was likely not an influential factor in 2SLGBTQQ+ IPV experiences and (2) consideration of parenthood status of 2SLGBTQQ+ individuals was beyond the scope of this study. Four team members (SK, SM, JN, OG) conducted the interviews. To increase comfort and safety, participants received the interview guides prior to the interview date, were asked if they had a preference of interviewer gender, were encouraged to take breaks as needed, and were reminded to share information based on their personal comfort level during the interview. We offered participants an opportunity to debrief after the interview and provided a list of no-cost support services should they need additional support once the interview finished. Interviewers also asked participants to avoid using names of their ex-partner’s, family members, etc. . Participants received a cash honorarium ($40 CDN) for their participation in the study.

Handheld audio recorders permitted audio recording of interviews conducted by phone and Zoom; however, we also recorded and downloaded Zoom interviews. Immediately after, we uploaded audio recordings to a secure drive protected by a two-factor password encryption system and promptly deleted them from recording devices. We initially manually transcribed interviews; however, ascertainment of ethics approval for the use of Otter.ai (online platform) permitted artificial intelligence to transcribe the remaining interviews (*n* = 17/46).^28^ At least one team member reviewed each Otter.ai completed transcript to ensure accuracy. We assigned an identification number to each transcript to maintain organization while ensuring anonymity.

### Data analysis

Once all interviews were complete and transcribed, three team members undertook thematic analysis of the interview data^23,24^; two authors completed initial analysis (JN, SM) and a third author reviewed all interviews (SK). To facilitate data analysis, all interview transcripts were uploaded onto Dedoose,^29^ a web application developed mainly for qualitative and mixed-methods research that helps to organize and analyze uploaded data. Dedoose was chosen as it is a user-friendly program which allows for large qualitative datasets, such as the one used in this study, to be easily analyzed and coded by multiple team members.^29^ Though shame-to-guilt behaviors were apparent in the initial data collection period, we did not confirm shame-to-guilt behaviors as the central concept of this article until we completed all data collection phases. Once the three authors (SK, JN, SM) reviewed the interviews and became more familiar with the content, they developed codes to either categorize codes into one major theme, divide them into separate themes, or develop new themes.^30^ Smaller codes were often grouped together to develop more prominent themes. This approach permitted identification of patterns, themes, and subthemes and encouraged reproducibility.^30^ Overall, inductive approaches guided initial analysis to derive shame-to-guilt behaviors during and following the end of the relationship, while deductive approaches allowed for subthemes to be identified and grouped as a function of the derived themes. Perspectives from service providers were incorporated into themes to discuss how professionals perceived and approached such experiences. Data saturation was achieved by iteratively examining the interviews and after analyzing all interviews. Less recurrent themes (i.e., themes 6 and 7) were included despite their infrequency as they are still important findings and can serve as areas for future research.

## Results

Demographic information is provided to characterize the sample of individuals who experienced IPV (*N* = 29; Table 2). In both groups, most of the participants identified their race as White/Caucasian (9 rural women and 11 2SLGBTQQ+ participants). All of the interviewed rural women described their experiences of IPV in the context of heterosexual relationships. 2SLGBTQQ+ participants identified their sexual orientation as primarily lesbian or bisexual (*n* = 9) and their gender as “female” (*n* = 6) or trans (*n* = 4). Ages for rural women participants ranged from 23 to 66 years with a mean of 41.73 years (SD = ± 14.13) and for 2SLGBTQQ+ individuals ages ranged from 20 to 43 years with a mean of 28.94 years (SD = ± 6.99). Most 2SLGBTQQ+ and rural women participants had some post-secondary education with moderate income levels (under $60,000). Lastly, most rural women had three or more children. All the rural women interviewed described experiencing aspects of physical and emotional or psychological abuse. Financial abuse (*n* = 9), sexual abuse (*n* = 6), and stalking (*n* = 7) were also significantly experienced by this group. Some rural women participants described spiritual abuse (*n* = 3) and the denial of medical needs (e.g., denied access to healthcare; *n* = 2). Physical (*n* = 11) and emotional/psychological abuse (*n* = 16) were described among 2SLGBTQQ+ individuals interviewed, with sexual abuse (*n* = 10), stalking (*n* = 6), and financial abuse (*n* = 5) also being prominently described.

**Table 2.** Participant demographics (*N* = 29).

Demographic variable	2SLGBTQQ+ (*n* = 18)	Rural women (*n* = 11)
Age (years), mean ± SD, range	29 ± 7, 20–43	42 ± 14, 23–66
Race (%)		
White	61	91
Asian	11	0
Indigenous	17	0
Biracial	6	0
Hispanic	0	9
Not answered	6	0
Sexual orientation (%)		N/A (all rural women described their relationship in the context of a cisheterosexual relationship)
Lesbian	28	
Gay	6	
Bisexual	28	
Pansexual	6	
Other^a^	33	
Gender (%)		All self-identified as a ciswoman
Female	22	
Male	6	
Transman/male	11	
Transwoman/female	11	
Cis/cisgender/cisgender female	11	
Two-spirit	6	
Genderfluid/queer	11	
Other^a^	22	
Children (%)	N/A (not asked)	
0		36
1		9
2		0
3		18
4		18
5		18
Education (%)		
High school	0	18
Some college or undergraduate school	39	0
Undergraduate degree or college diploma	56	82
Graduate degree	6	0
Annual household income in Canadian dollars (%)		
$0 to $24,999	33	36
$25,000 to $59,999	28	18
$60,000 to $99,999	22	9
$100,000 to $200,000	11	18
Not answered/did not know	6	18
Employment status (%)		
Unemployed	28	45
Student	6	0
Employed part-time or contract	17	18
Full-time	50	36

SD: standard deviation.

a Includes a mix of two or more sexual orientations or gender, respectively.

We also provide service provider information (*N* = 24). Service providers serving 2SLGBTQQIA+ (*n* = 8) were employed by agencies involved in shelters (*n* = 2), a police-based service (*n* = 1), a sexual assault center (*n* = 1), and agencies providing a range of direct support services to 2SLGBTQQ+ individuals (*n* = 4). All the service providers serving 2SLGBTQQ+ communities worked in director or coordinator roles and had been in those roles for 5 years or less. Service providers serving women in rural communities (*n* = 16) were employed by agencies involved in providing a range of direct support services (*n* = 9), shelters (*n* = 4), provincial organization of sexual assault centers (*n* = 1), municipal community development (*n* = 1), and a police-based organization (*n* = 1). Service providers serving rural women served in director, coordinator, or manager roles (*n* = 8), counsellor or outreach roles (*n* = 6), an administrative role (*n* = 1), or as a pastor (*n* = 1), providing direct support to rural women experiencing IPV. The years in their role ranged from less than 1 year (*n* = 6) to more than 10 years (*n* = 2).

Findings regarding shaming-to-guilt behaviors are categorized under seven themes, including shaming identity in relation to gender and sexual orientation, emotional and sexual manipulation, threats of death by suicide, apologies and vacuous promises as components of the cycle of abuse, using one’s parenting and children’s well-being to manipulate partners, the use of health conditions and faking illness, and the use of religion or faith to reinforce gender standards. All quotes which support the identified themes of this study are provided (Supplemental Material Table B).

### Theme 1: shaming identity in relation to gender and sexual orientation

This theme encapsulated behaviors that abusers used to shame their partners based on their gender and sexual orientations. Participants from both rural women (*n* = 3) and 2SLGBTQQ+ (*n* = 3) groups who experienced IPV described how gender-constructed standards were used to lower their self-esteem, limit their choices, and suggest they are not trustworthy. This is captured well by the following rural woman who experienced sexual abuse: “so then it turned into sexual abuse where he demanded that I perform my wifely duties, which was whatever he wanted and whenever he wanted. . . And if I did anything to step out of line, he would use it against me” (RW P1). For rural women specifically, there were emerging patterns of being shamed and labeled as unfaithful when going out with friends or spending time with others, being “accused of doing, you know, like [unfaithful] things” (RW P8) or told “oh you’re just gonna go on this trip and be a hoe” (RW P11) to coercively guilting them into isolation. One service provider also described examples of prescribed gender roles by stating that the “wife does what [the] husband says to do and often have to ask if they can use the vehicle or go places. . . they have to stay home and do the housework. . . stay home because our baby cries too much. . . ‘Your body is my body and we will have sex when I say so. . . ‘[the abusive partner] decides what happens with [their] money’” (RW SP5D). Such beliefs stem from patriarchal beliefs which undermine women’s engagement in society and the labor market.

Two 2SLGBTQQ+ individuals experienced invalidation of their sexual identity during the relationship (e.g., “every time we got into a little bit of an argument, she would bring up my transgender state. . . which was mentally exhausting for me” (2SLGBTQQ+ P6)) and postseparation (e.g., “let me guess you have a boyfriend now or something. . . you were never actually like bisexual, pansexual, you’re just like faking it for attention” (2SLGBTQQ+ P17)). Furthermore, threatening to “out” one’s sexual orientation, or to disclose one’s sexual orientation to others who were unaware of their identity, was also unique to a 2SLGBTQQ+ participant: “a lot of the times when she wasn’t happy with what I did, she would threaten by saying that she would out me to my parents” (2SLGBTQQ+ P16). Three service providers reinforced this fear of outing, as reflected by the following statement: “we hear a lot of one partner threatening to ‘out’ another partner” (2SLGBTQQ+ SP5).

### Theme 2: emotional and sexual manipulation

Emotional manipulation manifested in both 2SLGBTQQ+ (*n* = 7) and rural women (*n* = 3) groups, involving behaviors such as “gaslighting” whereby abusers distort their partners perceived realities, manipulating them into not trusting their own perceptions and decreasing their confidence. Gaslighting behaviors involved comments such as “why can’t you just take a joke” (RW P11), “I told you so” (RW P10), “don’t you love me?” (2SLGBTQQ+ P2), and “he basically told me. . . I was crazy, said that I was bipolar and that I was making everything up in my head” (2SLGBTQQ+ P9). Gaslighting also extended post-separation. For example, one rural woman participant described how she needed to talk to her ex-partner at their daughter’s birthday because “there was something that [she] didn’t agree with, [she] didn’t like the way something was being handled,” but the conversation turned into “a me-thing, that I’m the problem here, look I just need to do his thing, he’s wanting to take my daughter out, I should feel lucky, all of this stuff” (RW P1). Shaming an individual’s independence was an outcome that occurred for a 2SLGBTQQ+ participant, whereby the abusive partner “shamed and guilted [them] for choosing to live alone and support [themselves] and pay [their] bills instead of living with [the abusive partner]. . . And so, [they were] shamed and guilted for basically choosing to live alone or having [their] own independence” (2SLGBTQQ+ P8).

Service providers further (*n* = 3) described how abusers may use comments to make their partners believe nobody else will want them: “when somebody’s been telling you that you’re nothing, you have nothing, nobody will do anything for you and you’re not worthy, that, you know, the fear of, knowing okay, well at least I have this person” (2SLGBTQQ+ SP4). Another described the implications of abusers’ destruction of their partners’ self-esteem on help-seeking by stating that “you know, because they’ve been told they’re not good enough, they don’t know how to do things, they don’t feel that, they can even, like what’s the point of even asking for help” (RW SP8). Another service provider also discussed the fear of being shamed by the broader 2SLGBTQQIA+ community when in an abusive relationship especially if “the partner [is] a prominent member of the community, they are afraid of what other people might think or say, maybe depending on the status of affluence that [the abusive partner] has” (2SLGBTQQ+ SP8).

Individuals experiencing abuse in both the 2SLGBTQQ+ (*n* = 2) and rural women (*n* = 2) groups expressed how abusers used emotional manipulation to guilt them into performing sexual activities as it was expected of them to engage in sexual intercourse. One rural woman described how her abusive ex-partner “would have tantrums about like sexual stuff, if he didn’t get sex or he didn’t get oral sex. . . it was easier to just get it over with rather than him throwing a fit for three days” (RW P9). Similarly, another participant stated how they would end up “giving in cause they won’t stop” (2SLGBTQQ+ P15). Another rural woman also described how she would “be guilted into doing lots of things” (RW P8), particularly sexually, as ways to apologize when her abusive ex-partner was mad at her. One 2SLGBTQQ+ individual noted that a third individual who was emotionally engaged with their abusive partner through polyamory tried to trick them into a threesome, lying “to [the participant] about what [their] partner wanted” (2SLGBTQQ+ P3) to guilt them into doing it.

### Theme 3: threats of death by suicide

Rural women (*n* = 2) and 2SLGBTQQ+ individuals (*n* = 5) described how threats of death by suicide were weaponized by abusers to guilt them into remaining in the relationship or to prevent help-seeking, with many 2SLGBTQQ+ individuals alluding to this shame-to-guilt behavior. One rural woman described how the ex-partner “did once hold a gun to his own head and threaten to kill himself, which I suppose swayed my decision to seek help because then I was convinced I was the perpetrator and I was doing this to him” (RW P8) and a 2SLGBTQQ+ individual stated that “it was really hard to leave him, every time I tried to leave him he would threaten suicide” (2SLGBTQQ+ P9). Another rural woman participant described how her abusive partner leveraged her son’s death by suicide to have her return to the relationship: “[the partner] actually used the suicide from my son. . . And then he told me, just all the ways he was going to [engage in death by suicide]. . . he said it would involve people, and I’m like. . . you can’t place this on me cause you knew my son committed suicide. So, he knew where to get me with certain things” (RW P3). Rural women service providers (*n* = 2) also described how abusers would use threats of suicide to coercively control the individual experiencing abuse, stating how the “thought of having someone die because of you, your choices, that’s absolutely overwhelming” (RW SP5). These guilt inducing actions, as validated by the sample, seem to deepen the individual’s shame and the traumatic bond with the abuser, causing the individual to feel guilty, trapped, and insignificant.

### Theme 4: apologies and vacuous promises as components of the cycle of abuse

Apologetic and empty promises, aspects of the cycle of abuse, emerged as a theme more evidently among women living rurally (*n* = 5), although one 2SLGBTQQ+ participant described an experience related to this theme. More specifically, abusers leveraged the remorse or compassion that their partners experienced when apologizing or engaging in empty promises, which in turn, would induce feelings of shame-to-guilt.^31^ For example, a rural woman described how her abusive partner would ask “what could he do to get me, for me to come back to him, and he said to me, I will do counseling” (RW P2), making her feel remorse for them. Another described how their partner would return “with the gaslighting and the love-bombing and apologies and all that stuff, and I was still in love with him and all that” (RW P9), while another described that “the silent treatment started, [while also] the interjection of massive gifting and attention” (RW P10). A 2SLGBTQQ+ participant demonstrated this cycle in a longer-term scope, having occurred much later following the end of a restraining order: “two or three weeks after the restraining order expired, she actually sent me an email. . . telling me all about her life and asking if we could be in contact again. If she could see my child and she feels like she would really benefit from it and she would, you know, grow to appreciate [their] ex in her life again” (2SLGBTQQ+ P8). The abusive partner in this context attempted to emotionally guilt the partner into contact again by describing how they have changed and how positive it would be for them to reconnect. A service provider supported this notion, describing what follows an outburst event, where the abusive partner “will then try to get you back into that honeymoon stage. So they will groom you to believe that that was a one off” (RW SP2), attempting to make individuals feel remorse for the partner through false stories. Overall, the use of promises, apologies, or kind remarks triggers sentiments of guilt and remorse in the abused partner, sometimes dissuading them from leaving the abusive relationship or to return to it.

### Theme 5: using one’s parenting and children’s well-being to manipulate partners

Three rural women described shame-to-guilt behaviors in the context of parenting, such that parental responsibilities and children were used by abusive partners in coercive manners to maintain dominance over their partners. A rural woman participant described how her abusive ex-partner targeted her ability to parent to make her feel ashamed, guilty, and then less confident in her capacity as a parent, “expecting [them] to parent [their] children the way he wanted [them] to parent [the children]. . . I thought it was a problem with me, so I thought I was growing and learning, but in hindsight I can see that what he was asking and telling me to do was an extremely unhealthy parenting attitude” (RW P1). The abuser further shamed this participant for behaviors such as self-injury rather than providing support, telling the participant that she was not capable of parenting if she was self-harming. Furthermore, the fear of losing custody of their children was also an impediment to leaving or seeking help for the IPV. One rural woman stated that she was told “many times that I couldn’t, if I left, I wouldn’t be able to have the kids, and so I thought, there’s no way I’m leaving my kids with you” (RW P2), feeling shamed-to-guilted to stay in the abusive relationship through her responsibility and desire to be a parent. Another participant described how her abusive partner would state that he “miss[es] [their] kids” as an attempt to guilt his ex-partner into contact again (RW P11). Service providers from both groups described shame-to-guilt behaviors based on parenting. One service provider described how the individual experiencing abuse may “want to keep their family together” because “they don’t love the behavior, but they love their spouses” and “their ability to blame themselves is unbelievable, and their partner reinforces it for them regularly” (RW SP2).

### Theme 6: the use of health conditions and faking illness

Participants described the use of health conditions by their partners as a means to try to induce shame-to-guilt. Service providers pointed out that abusers might make “people feel shameful about some of the medical care that they need in order to be themselves” specifically in the context of trans individuals (2SLGBTQQ+ SP3). Another service provider described that they are “starting to see individuals that are transitioning and we’re hearing more about how the medication [and hormones are] affecting their ability to eat. But then again with [the IPV] and [the] anxiety and depression it’s sometimes a struggle to eat, or you know, so there’s those disordered eating [that can emerge]. But [there’s] also obesity, sometimes with the gaining of the weight, and then the shame that the perpetrator is putting on them around that” (2SLGBTQQ+ SP7). A service provider touched on the implications of COVID-19, stating “that COVID-19 has been absolutely weaponized by abusers in several facets. So one, it’s like, you can’t go to that hospital, because if you do, you’re gonna get COVID. And so that was kind of the most common thing that we saw” (RW SP5).

In both groups, faking medical illness was used by an abuser to incur feelings of shame-to-guilt, gain control over their partners, and ensure that their partners were catering to their unfounded needs. One rural woman and two 2SLGBTQQ+ individuals described this, as reflected by the following two quotes: “he actually faked a disability so he stopped working shortly after we got married too and put a huge financial burden on me. So, I had to work fifty or sixty hours a week to make ends meet because he just up and quit working. . . If I asked him to do a load of laundry or like a load of dishes, it was the end of the world. . . He would guilt trip me because he’d be like ‘I’m disabled and you’re abusing me’, like ‘You’re an abusive caretaker’, that kind of thing.” (RW P9) and “[she] emotionally manipulated me into believing that she had stomach cancer. So that was a whole play on my caring nature. . . [it] really affected how well I took care even of people in my family. So with that one I lost my job, I lost my home, I lost everything at the end of that relationship” (2SLGBTQQ+ P7).

### Theme 7: the use of religion or faith to reinforce gender standards

Though less common, this theme refers to shame-to-guilt behaviors that abusers engaged in through the use of religion or faith to reinforce traditional gender standards. A 2SLGBTQQ+ individual (*n* = 1) and rural woman (*n* = 1) touched on their abusive ex-partner’s use of religion to establish how the individual experiencing abuse should have acted in the relationship, incurring feelings of shame-to-guilt when not aligning to certain gender standards. For one rural woman, claiming Christianity ended up in “lots of twisting of scripture and guilt, shame, and condemnation” given how this religion emphasized the role of a woman (RW P1). A 2SLGBTQQ+ individual stated how the ex-partner got them “involved in a church that. . . just basically God help me to all these standards” (2SLGBTQQ+ P9), using religious standards to shame-to-guilt the participant based on their sexuality and gender identity.

## Discussion

This qualitative study aimed to describe shame-to-guilt behaviors that were used in abusive relationships among 2SLGBTQQ+ and rural women groups as mechanisms of coercive control even after the relationship ended. First, identity-based tactics such as attacks on gender and sexual identity were used to shame-to-guilt partners, predominantly occurring among 2SLGBTQQ+ individuals. Emotional and sexual shame-to-guilt behaviors were also used as means to coercively control partners. Abusers further applied threats of death by suicide and faked medical illnesses to shame-to-guilt their partners. The use of parenting, children, and religion were also identified as means through which abusers shame-to-guilted their partners, particularly among women living rurally. Overall, 2SLGBTQQ+ individuals and rural women situated shame-to-guilt behaviors within a manipulative context that involved abusers engaging in disingenuous apologetic behaviors following outburst events as an avoidance method against permanent intimate partner relationship dissolution.

### Prominent themes for 2SLGBTQQ+ individuals

2SLGBTQQ+ individuals and service providers situated emotionally manipulative behaviors within the realm of unique mental health challenges experienced by sexual minorities. Mongelli et al.^32^ conceptualized Meyer’s^33^ minority stress model in order to elucidate the stressors experienced by 2SLGBTQQ+ individuals as a result of the societal stigma and discrimination that they experience. This, in turn, contributes to higher levels of psychological distress and shared trauma,^34^ which abusers can use strategically to coercively control their partners. Additionally, outing is another area that is targeted to induce shame-to-guilt. Outing entails “exposing someone’s lesbian, gay, bisexual, transgender, or gender non-binary identity to others without their permission. Outing someone can have serious repercussions on employment, economic stability, personal safety or religious or family situations.”^35^ Abusive partners can leverage their partner’s fear of outing to maintain control and power over them, a process which may be even more effective among older populations whom have hidden their sexual or gender identity for a lengthy period of time and who may be less likely to seek support from formal services.^36^ Synonymous with previous research, the above examples by which abusers leveraged the precarious mental health situation and identities of their partners in this study included tactics aimed at the diminishment of self-esteem,^37^ gaslighting,^38^ and sexual coercion.^39^ The importance of effective service provider responsiveness to shame-to-guilt emanating from experiencing IPV is thereby accentuated.^40^

### Prominent themes for women living rurally

For women living rurally, abusers’ use of the parenting context to strategically shame-to-guilt their partners was observed. By shaming a mother’s capacity to provide care, they may start to question their capacity as a parent, and further, to worry about child protection services from becoming involved. This phenomenon is captured well by Heward-Belle,^41^ who speaks to the narrative around the “good mother” and how preconceived or forced perceptions of parenting enable abusers to employed diverse coercively tactics to control their partner. Traditional expectations emphasizing how mothers should remain at home with their children or cater to their husband’s needs further convolutes the notion of good parenting.^41,42^ These ideas are typically exacerbated within rural settings in which patriarchal families are more prevalent and where families are larger (e.g., six of eleven rural women had three or more children).^42^ Such an interplay is even further complicated when the abuser is in a position of power, advantageously utilizing this to manipulate those in formal support roles (e.g., police) into limited action when the individual experiencing abuse reports occurrences of abuse.^21^ Intersections with parenting are further highlighted when using children as points of control. Abusive partners may tell false stories to children in attempt to shame their partners or guilt partners into remaining in the relationship through “leave reach” of the children,^43^ a term that reflects how abusive partners can manipulatively reach their partners by threatening to leave with the child(ren). Service providers supporting rural women also vocalized these concerns. To support women living rurally in understanding how their abusive partners may be utilizing parenting to control them, providers and healthcare professionals should describe to mothers what this may manifest as; mothers may then be able to self-identify their experiences, be relieved of the shame put upon them, and seek further help.

From a broader perspective, several rural women participants described their experiences of shame-to-guilt through apologies and vacuous promises as components of the cycle of abuse. These individuals described how the abusive partner would return with apologetic and kind behaviors to attempt to reconnect following separation, which would lead to confusion and the potentiality for re-entering the relationship. Such experiences illustrate exactly that which often fuels the cycle of abuse and reinforce how theory occurs in actuality.^5^ As geographically isolated individuals experiencing IPV encounter many challenges in leaving the abusive relationship apart from the partner’s abusive behaviors (e.g., financial implications, housing),^44^ they should be provided with social, mental health, and welfare services to reduce the impacts of the abusive partner’s cyclically abusive behavior and increase the likelihood for successfully leaving the relationship.

### Abusers’ use of shame-to-guilt in both groups

Shame-to-guilt behaviors in relation to gender and sexual orientation intersected differentially between the 2SLGBTQQ+ and rural women groups. In relation to gender and sexual identity, rural women emphasized that they were guilted by the need to perform “wifely duties.” This is likely the embodiment of a patriarchal way of living that some cisheterosexual males maintain in their relationships, stemming from gender constructs and norms.^11,45^ Other studies corroborate this way of thinking, where males who maintain traditional perspectives that women should be hyperfeminine are more likely to be abusive.^45,46^ In comparison, a 2SLGBTQQ+ individual described identity invalidation and threats of being outed (which was further corroborated by service providers), paralleling other research.^47–49^ Overall, these findings emphasize the importance of attentiveness to identity manipulation as a means by which abusers of IPV may engage in shame-to-guilt behaviors.

In accordance with previous research,^50–52^ 2SLGBTQQ+ individuals reported that their abusers used threats of death by suicide to shame-to-guilt them into remaining in the abusive relationships; however, rural women also reported such behaviors. Given the gravity and sensitivity of death by suicide, abusers can strategically control their partners and manipulate this interaction to their benefit. Whether abusers actually intend death by suicide or not, it is not the ex-partner’s responsibility to provide support to the abuser; however, abusers can use this feeling of responsibility to shame-to-guilt their partners, demonstrating another approach to coercive control. This is supported by a study by Fitzpatrick et al.,^53^ which demonstrated how threats of suicide can be tactically used to manipulate those experiencing IPV. This alarming approach to coercive control is intrinsically linked to mental health challenges,^53^ requiring attention from providers to reduce the burden that individuals experiencing IPV may feel when encountering such experiences during or following the end of the intimate partner relationship.

The use of religion to manipulate and coercively control intimate partners was described by one participant in each group. Though uncommon in this study, the impacts of such shame-to-guilt were profound. Abusive partners invoked religion to shame-to-guilt their partners and manipulated their partners into affiliating with the abusers’ religious communities or churches to further control their partners. For individuals who practice a faith, their religious community may be privy to the abuse as well as one of the first places that individuals experiencing IPV turn to for help.^54^ In this study, religious affiliation was also seen to impact help-seeking behaviors. For one rural participant, their relationship with a pastor and church (not affiliated with her abusive partner) was a source of valuable support. However, for another rural participant, affiliation with their abusive partner’s religious community was used to further shame and limit the women’s help-seeking efforts. As religion can be a sensitive topic to discuss, providers should undergo training and seminars on how to approach religion in the context of IPV, whether used as a form of abuse or as a help-seeking avenue, particularly to sensitively undo or shift one’s belief of what is owed to another person.

Less commonly, abusers lied about medical illnesses and used COVID-19 to their advantage to coercively control their partners. Though manifesting in different manners, all these abusive behaviors had a goal of maintaining dominance over the individual experiencing IPV. Abusers can use fake illnesses advantageously to acquire specific forms of attention and care from their partner.^55^ This is a phenomenon described in the DSM-5, termed factitious disorder.^55^ COVID-19 was also used by abusers to obtain a desired outcome (e.g., physical presence) by incurring feelings of guilt among their partners through descriptions of following lockdown guidelines and health implications. Ensuring that providers are aware of these potential manifestations of shame-to-guilt is imperative to educate those experiencing IPV and provide different avenues of support.

### Policy and practice implications and recommendations

Four key recommendations arose from the findings of this study in addressing shame-to-guilt: education, service provider practices, community support and safe spaces, and enhancing the justice system. First, given the extensive comments participants made about their identity and sexual preferences being attacked and how it induced shame-to-guilt, it would be advisable that psychoeducational material on emotional abuse/coercive control include specific references on how sexual and gender identity can be used as a weapon to manipulate, shame, and control individuals experiencing IPV. These resources should be integrated to promote awareness among: (1) teachers of children beginning at a young age to raise awareness of this form of shame-to-guilt behavior for 2SLGBTQQ+ individuals entering intimate partner relationships; (2) policymakers and government bodies to promote additional research in this area, knowledge mobilization, and resource and service development; (3) frontline service providers to make sure 2SLGBTQQ+ individuals experiencing such forms of IPV are provided with appropriate support; and (4) school systems in rural settings to deconstruct patriarchal teachings and understandings that often start from a young age so that not only are girls/women encouraged to contribute equally within society but also that boys/men avoid learning power imbalances and gender stereotypes that can be conducive to later engaging in gender-based IPV.

Furthermore, service providers need to continue to be integrated into the 2SLGBTQQ+ community by sharing specific examples of abuse that are relevant to this population and pointing out that abuse is abuse regardless of one’s gender and sexual orientation. Given that many of our participants shared that they doubted themselves via shame-to-guilt inducing behaviors, it is imperative for service providers to give extra attention to providing choice to their clients. This can be done by having a very clear consent process and providing options of possible services rather than directing clients into action. Once safety has been established, therapeutic work with 2SLGBTQQIA+ individuals experiencing IPV like others experiencing IPV may benefit from trauma-informed care to reduce the shame-to-guilt impact. This may include trauma processing (e.g., eye movement desensitization and reprocessing^56^) and life skills to reclaim their inherent right of having their boundaries honored and protected. Assertiveness and self-esteem work may help heal the wounds shame-to-guilt behaviors may have caused.

Targeted awareness of society’s dismissal and normalization of abuse along with education on emotional and psychological abuse is important in the context of coercive control, guilt-tripping, and gaslighting for rural communities and 2SLGBTQQ+ individuals. For example, an educational curriculum (e.g., sex and health class) designed to be more inclusive of diverse sexual and gender identities could support 2SLGBTQQ+ individuals as emotional forms of abuse within 2SLGBTQQ+ relationships can diverge from those observed among cisheterosexual relationships. Overall, the cycle of abuse needs to be expanded to showcase how triangulation and shame-to-guilt are used to manipulate and control individuals experiencing IPV within specific identity groups, such as rural women and 2SLGBTQQ+ groups.

Lastly, changes in the justice system could also be of benefit to include other forms of shame-to-guilt such as uttering threats of death by suicide to coercively control those experiencing IPV into remaining in the relationship and not seeking help, highlighting the importance of coercive control in legislation. In Canada, for example, Bill C-202 enactment amends the Criminal Code section 264.1 “to create an offence of engaging in controlling or coercive conduct that has a significant impact on the person towards whom the conduct is directed, including a fear of violence, a decline in their physical or mental health.”^57^ However, an elaboration of what this entails is needed to better understand what is considered a significant impact on day-to-day activities (and who is subject to making this decision, particularly in relation to mental health) and to promote transparency for education and awareness.

### Limitations and strengths

Despite attempts in objective completion of interviews and data analysis, we are cognizant of the role of reflexivity and the potential effects that personal values and experiences may have had on the findings. However, to limit this, we did meet repeatedly and discussed why data were coded to reach a more objective consensus. Furthermore, this study used data from interviews that did not directly ask questions pertaining to shame-to-guilt, decreasing the potential saturation of findings that may have arisen. Had we asked direct questions specific to shame-to-guilt, we would have likely obtained more responses. Nevertheless, these experiences arose of participants’ narratives naturally, suggesting the commonality and worthiness to report on such experiences. Lastly, the experiences of individuals who experienced IPV may not reflect the experiences of all individuals experiencing IPV as many do not disclose violence or take part in research projects. Furthermore, IPV experience may also differ based on other intersecting identities (e.g., race, disability) which provide abusers with other means of shaming-to-guilt partners. However, our 2SLGBTQQ+ group did consist of a diverse group of individuals.

## Conclusion

This study investigated how shame-to-guilt manifested as forms of IPV within 2SLGBTQQ+ and rural women communities. There were overlapping and unique mechanisms by which participants from each community experienced shame-to-guilt as forms of coercive control. Some behaviors included shaming one’s independence, faking medical illness, and the use of COVID-19. Furthermore, 2SLGBTQQ+ individuals and rural women highlighted their subjugation to disingenuous apologetic behaviors from abusers as a preventive method against permanent relationship dissolution. Dominant mechanisms by which 2SLGBTQQ+ individuals experienced shame-to-guilt encompassed invalidation of gender and sexual identity and preying on participants’ mental health. Rural women placed emphasis on apologies and vacuous promises as components of the cycle of abuse in their narratives and on familial dynamics including parenting and children’s well-being. In closing, it is anticipated that future research which investigates IPV within larger samples of 2SLGBTQQ+ and rural women communities will further elucidate the mechanisms by which shame-to-guilt behaviors are experienced by sexual and gender minorities and those living in geographically isolated locations, respectively, to support individuals in successfully leaving the abusive relationship. Shaming is a strategic technique that abusive partners impose on their partners to incur feelings of guilt (i.e., shame-to-guilt); untangling how this occurs is imperative to support individuals feeling guilty to escape the degradative relationship and seek healthier relationships.

## Supplemental Material

sj-docx-1-whe-10.1177_17455057251335361 – Supplemental material for “Don’t You Love Me?” Abusers’ use of shame-to-guilt to coercively control 2SLGBTQQIA+ individuals and rural women experiencing intimate partner violence Supplemental material, sj-docx-1-whe-10.1177_17455057251335361 for “Don’t You Love Me?” Abusers’ use of shame-to-guilt to coercively control 2SLGBTQQIA+ individuals and rural women experiencing intimate partner violence by Stefan Kurbatfinski, Nicole Letourneau, Jason Novick, Susanne Marshall, Keira Griggs, Dawn McBride and Kendra Nixon in Women’s Health
