# Supplementary material for: “Don’t You Love Me?” Abusers’ use of shame-to-guilt to coercively control 2SLGBTQQIA+ individuals and rural women experiencing intimate partner violence
Source: Womens Health (Lond). 2025 Apr 30;21:17455057251335361. doi: 10.1177/17455057251335361 (PMC12046170; doi:10.1177/17455057251335361)
Supplement: sj-docx-1-whe-10.1177_17455057251335361 – Supplemental material for “Don’t You Love Me?” Abusers’ use of shame-to-guilt to coercively control 2SLGBTQQIA+ individuals and rural women experiencing intimate partner violence [file sj-docx-1-whe-10.1177_17455057251335361.docx]

**Table A.**

*Consolidated Criteria for Reporting Qualitative Research (COREQ) guidelines*

| **Item No** | | **Guide Questions/Description** | **Reported on Page #** |  |
| --- | --- | --- | --- | --- |
| **Domain 1: Research team and reflexivity** | | | |  |
| **Personal Characteristics** | | | |  |
| 1. Interviewer/ facilitator | | Which author/s conducted the interview or focus group? | Pg 9 |  |
| 2. Credentials | | What were the researcher’s credentials? E.g., PhD, MD | Pg 1 & 9 |  |
| 3. Occupation | | What was their occupation at the time of the study? | Pg 9 |  |
| 4. Gender | | Was the researcher male or female? | N/A |  |
| 5. Experience and training | | What experience or training did the researcher have? | Pg 9 |  |
| **Relationship with participants** | | | |  |
| 6. Relationship established | | Was a relationship established prior to study commencement? | Pg 8 & 9 |  |
| 7. Participant knowledge of the interviewer | | What did the participants know about the researcher? e.g. personal goals, reasons for doing the research? | Pg 9 |  |
| 8. Interviewer characteristics | | What characteristics were reported about the interviewer/facilitator? e.g. Bias, assumptions, reasons and interests in the research topic | Pg 28 |  |
| **Domain 2: study design** | | |  |  |
| **Theoretical framework** | | |  |  |
| 9. Methodological orientation and Theory | What methodological orientation was stated to underpin the study? e.g. grounded theory, discourse analysis, ethnography, phenomenology, content analysis | Pg 7 |  |  |
| **Participant selection** | | |  |  |
| 10. Sampling | How were participants selected? e.g., purposive, convenience, consecutive, snowball | Pg 8 |  |  |
| 11. Method of approach | How were participants approached? e.g., face-to-face, telephone, mail, email | Pg 8 |  |  |
| 12. Sample size | How many participants were in the study? | Pgs 10 & 11 |  |  |
| 13. Non-participation Setting | How many people refused to participate or dropped out? Reasons? | N/A |  |  |
| 14. Setting of data collection | Where was the data collected? e.g., home, clinic, workplace | Pg 6 |  |  |
| 15. Presence of nonparticipants | Was anyone else present besides the participants and researchers? | N/A |  |  |
| 16. Description of sample | What are the important characteristics of the sample? e.g. demographic data, date | Pgs 8 & 9 |  |  |
| **Data collection** | | |  | No |
| 17. Interview guide | Were questions, prompts, and guides provided by the authors? Was it pilot tested? | Pg 8 and Supp Mat |  |  |
| 18. Repeat interviews | Were repeat interviews carried out? If yes, how many? | N/A |  |  |
| 19. Audio/visual recording | Did the research use audio or visual recording to collect the data? | Pgs 8 & 9 |  |  |
| 20. Field notes | Were field notes made during and/or after the interview or focus group? | N/A |  |  |
| 21. Duration | What was the duration of the interviews or focus group? | Pg 9 |  |  |
| 22. Data saturation | Was data saturation discussed? | Pg 10 |  |  |
| 23. Transcripts returned | Were transcripts returned to participants for comment and/or correction? | N/A |  |  |
| **Domain 3: analysis and findings** | | |  |  |
| **Data analysis** | | |  |  |
| 24. Number of data coders | How many data coders coded the data? | Pg 10 |  |  |
| 25. Description of the coding tree | Did the authors provide a description of the coding tree? | N/A |  |  |
| 26. Derivation of themes | Were themes identified in advance or derived from the data? | Pg 10 |  |  |
| 27. Software | What software, if applicable, was used to manage the data? | Pg 10 |  |  |
| 28. Participant checking | Did participants provide feedback on the findings? | N/A |  |  |
| **Reporting** | | |  |  |
| 29. Quotations presented | Were participant quotations presented to illustrate the themes/findings? Was each quotation identified? e.g., participant number | Pg 13-20 and suppl mat |  |  |
| 30. Data and findings consistent | Was there consistency between the data presented and the findings? | Pg 13-20 and suppl mat |  |  |
| 31. Clarity of major themes | Pg 13-20 and suppl mat | Pg 13-20 and suppl mat |  |  |
| 32. Clarity of minor themes | Is there a description of diverse cases or a discussion of minor themes? | Pg 13-20 and suppl mat |  |  |

**Interview Guides.**

*Interview guide for rural women study.*

**I. Background/Demographics:**

1. Can you please tell me your age? __________ (in years)
2. Are you currently employed?
   1. No ______
   2. Yes – full time ______
   3. Yes – part time ______
   4. Yes – casual ______
3. What is the highest grade/level of education you have completed? __________________
4. What is your total annual household income (approximate)? ________________
5. With which ethno-cultural or racial group do you most identify? ­­­________________________

5a. Are you a newcomer or recent immigrant (came to Canada within the last five years)?

_____ Yes _____ No

1. 6a. What is the relationship status with your abusive partner/ex-partner?:
   1. Married ____ Separated/Divorced ____
   2. Common-law ____ Single ____
   3. Dating ____ Widowed ____
   4. Other (specify) ____

6b. Is this a same-sex relationship? Yes _____ No_____

1. Do you have children?

_____ Yes _____ No (if “No” skip to question 10).

1. If yes, how many? Ages?
2. Do your children live with you? Yes______ No________

9a. If yes, how many of your children live with you? ____________________

9b. If some of your children live elsewhere, where do they live?

[Check all that apply]

Father/Mother

Grandparents or other relative ______

Child welfare: temporary placement permanent placement ______

Children live on their own (adult children) __

Other (describe)

1. While you were in a violent/abusive relationship, were you responsible for the care of farm animals or livestock? Yes______ No________

**II. Nature of the Violence/Women’s Experiences of Abuse**

1. In whatever detail you are most comfortable with, can you please describe the nature of the violence/abuse you experienced at the hands of your partner/ex-partner?
2. Are you still in a relationship with this person?

2b. If not, when did your relationship end? How did your relationship end?

2c. Did you continue to experience abuse/violence after you separated?

3. Experiencing violence/abuse from an intimate partner can have profound impacts on women. How has your partner’s/ex-partner’s violence impacted

you?

Probes: Emotionally/psychologically, physically/medically, spiritually, financially, employment/school, relationship with others, relationship with

your children/mothering?

4. Do you believe living in a rural area/municipality has exacerbated (or worsened) these impacts? How so?

5. In addition to what you mentioned, are there other issues/challenges you are facing in your life that you believe are connected to your experiences

with abuse/violence and living in a rural area?

Probes: Addiction, physical disabilities, cognitive impairment, mental health issues, stress, etc.

6. Has the COVID-19 pandemic had an impact on your experiences with abuse/violence?

Probe: Please explain why/how this has occurred.

**III. Help Seeking Experience**

Many women (but not all) seek help for the violence perpetrated against them. We’d like to ask you some questions about your experiences of seeking help.

1. Have you ever told someone about the abuse you experienced by your partner/ex-partner?

Probes:

If no, why not?

If yes, who did you tell (i.e., family, friends, co-workers, neighbours, clergy/faith leader, professional)

- When was this?
- What made you decide to do this?
- Did living in a rural area effect your decision to disclose your experience with violence? How so?

1. Have you ever sought help from professionals as a way to address the violence perpetrated against you? If so, can you please tell me who you sought help from and what help you were hoping to receive?

Probes: Police/RCMP, Victim Services, shelter, lawyer, medical personnel, counsellor, crisis line, child welfare

E.g., to obtain physical safety, urgent medical attention, to recover/heal from physical and psychological stress, to stop the violence in the relationship

1. What was your experience with seeking help like?

Probes: What was your journey like (both positive or negative experiences)? Did you find their assistance helpful? Unhelpful? Why or why not?

4a. Seeking help can be very difficult for many women. Did anything make it difficult to disclose or seek help? What challenges did you face?

Probes:

- Isolation (including geographic)
- Access to technology (i.e., poor/unreliable/unavailable internet connection, cellular service)
- Transportation barriers (i.e., lack of transportation, expensive)
- Fear of losing your privacy, confidentiality? Fear of stigma, shame?
- Fear of racism/discrimination?
- Was it hard to find services?
- Fear of removal of children from CPS?
- Fear of retaliation, use of firearms?
- Care of pets, livestock, or family farm?
- Fear of financial insecurity/lack of housing?
- Fear of losing immigration status?
- Religious or faith-based considerations
- Parenting concerns
- Other challenges?

4b. How did living in a rural area impact your help-seeking or disclosing your experiences of abuse?

1. Were there other issues/challenges in your life that impacted your ability to seek help?

Probes: Financial, care of children/others, physical/mental health, sexuality or gender-diversity issues, immigration status, new to the community?

1. Has the COVID-19 pandemic impacted your ability to seek and/or receive help?

Probe: Please explain why/how this has occurred.

1. Did you have to leave your home community to access services or escape the abuse/violence? If so, what was this experience like for you and your children (if woman has children)?
2. Is there anything else you’d like to tell me about your experience disclosing the violence perpetrated against you or your experience seeking help?

Probes: with informal sources of help (E.g., friends and family)? With formal sources of help?

1. We’d like to know if you have any suggestions or recommendations for ways in which services for rural women who experience IPV could be improved. Can you please offer suggestions or recommendations?
2. Is there anything else that you would like to tell us about your experience of IPV? Is there anything else you think we should know?

**Thank you for taking the time for this interview and for sharing your thoughts and experiences. This can be a difficult topic to talk about and your responses are very valuable in helping us to understand the experience of rural women.**

*Interview guide for 2SLGBTQQIA+ study.*

**I. Background/Demographics:**

1. Can you please tell me the year you were born? ____________

2. What city/town do you reside in? _______________

3. What is your gender? _______________

Prompts: agender, man, woman, non-binary, trans, Two Spirit, another gender or additional gender identity.

4. What is your sexuality or sexual orientation? _______________

Prompts: asexual, bisexual, gay, heterosexual (straight), lesbian, pansexual, queer, questioning/unsure, Two Spirit, another or an additional sexuality/sexual orientation.

5. Are you currently employed?

a) No ______

b) Yes, full time ______

c) Yes, part time ______

d) Yes, casual ______

6. What is the highest grade/level of education you have completed? __________________

7. What is your total annual household income (approximate)? ________________

- 1. 8. Do you identify as having a disability and/or chronic health condition?
  2. a) _____ Yes _____ No
  3. b) If yes, please specify __________________

9. With which ethno-cultural or racial group do you most identify? ________________

a) Are you a newcomer or recent immigrant (came to Canada within the last five years)? _____ Yes _____ No

10. a) What is the relationship status with your abusive partner/ex-partner?

Married ____ Separated/Divorced ____

Common-law ____ Single ____

Dating ____ Widowed ____

Other (specify) ____

**II. Experiences of Intimate Partner Violence**

In this next section, we will ask you to discuss your experiences of intimate partner violence. When recounting your experiences of violence/abuse, please do not use any names of current or former partners.

11. In whatever detail you are most comfortable with, can you please describe the nature of the violence/abuse you experienced.

- 1. 12. Experiencing violence/abuse from an intimate partner can have profound impacts. How has your partner’s/ex-partner’s violence impacted you?
  2. Probes: Emotionally/psychologically, physically/medically, spiritually, financially, employment/school, relationships with others
  3. 13. Do you believe that your identification as a diverse gender identity and/or sexual orientation has impacted your experiences of intimate partner violence?
  4. Probes: forms of IPV specific to 2SLGBTQQ+ communities (i.e., threats of being outed, misuse of pronouns, not honoring chosen name, etc.). If yes, please describe how.

**III. Experiences Seeking Help**

Many victim/survivors (but not all) seek help for the violence perpetrated against them. We’d like to ask you some questions about your experiences of seeking help.

14. Have you ever reported your experiences of intimate partner violence to an informal support (i.e., friends, family, religious leader, etc.)?

a) If yes, describe your experience.

• If yes, did you find the assistance helpful? Unhelpful? Why or why not?

• Both positive and negative experiences?

• Did you feel safe/respected during these encounters?

b) If no, what prevented you from telling anyone about the violence or seeking help?

15. Have you ever reported your experiences of intimate partner violence to a formal support agency (i.e., police, victim services, legal service, shelter, etc.)?

- 1. a) If yes, describe your experience.

• If yes, did you find the assistance helpful? Unhelpful? Why or why not?

• Both positive and negative experiences?

• Did you feel safe/respected during these encounters?

• Was the agency/organization equipped to support victims/survivors from 2SLGBTQQ+ communities?

- 1. b) If no, what prevented you from telling anyone about the violence or seeking help?
  2. 16. Seeking help can be difficult for victims/survivors who identify with a 2SLGBTQQ+ community.
  3. a) Did your gender identity and/or sexual orientation impact your help-seeking or disclosing your experiences of abuse?
  4. b) Did anything else make it difficult to disclose or seek help?
  5. c) What challenges did you face?

Probes:

• Fear of stigma, shame?

• Isolation (including geographic)

• Access to technology (i.e., poor/unreliable/unavailable internet connection, cellular service)

• Transportation barriers (i.e., lack of transportation, expensive)

• Fear of losing your privacy, confidentiality?

• Fear of racism/discrimination?

• Fear of retaliation?

• Fear of financial insecurity/lack of housing?

• Fear of losing immigration status?

• Religious or faith-based considerations

• COVID-19 pandemic

• Other challenges?

- 1. 17. Have you ever been involved with the criminal and/or family court systems because of the intimate partner violence that you’ve experienced?
  2. a) If yes, describe your experience.
  3. b) Was the court sensitive to matters specific to 2SLGBTQQ+ communities (i.e., honoring chosen name, correct use of pronouns, etc.)?

18. Did you experience intimate partner violence during the COVID-19 pandemic?

a) If yes, what issues/challenges did this present in terms of your ability to seek help?

19. Do you have any suggestions or recommendations for ways in which services for members of 2SLGBTQQ+ communities who experience intimate partner violence could be improved?

**IV. Perceptions of Intimate Partner Violence**

- 1. 20. Do you perceive intimate partner violence to be a significant problem within 2SLGBTQQ+ communities?
  2. a) If yes, why?
  3. b) If no, why not?

21. Do you feel that the issue of intimate partner violence in 2SLGBTQQ+ communities is taken seriously? Probes: by professionals, by the broader community, by other members of 2SLGBTQQ+ communities?

- 1. 22. Have you encountered any myths/misconceptions surrounding intimate partner violence in 2SLGBTQQ+ communities?
  2. a) If so, where? From whom?
  3. b) In your opinion, what are the impacts of these myths/misconceptions?

23. Is there anything else you’d like to tell me about your experiences of intimate partner violence? Is there anything else you think we should know?

**Thank you for taking the time for this interview and for sharing your thoughts and experiences. This can be a difficult topic to talk about and your responses are very valuable in helping us to understand experiences of intimate partner violence in 2SLGBTQQ+ communities.**

**Table B.**

*Full list of quotes supporting identified themes.*

| **Subtheme** | **Quote** | **Community** |
| --- | --- | --- |
| **Theme 1: Shaming Identity in Relation to Gender and Sexual Orientation** | | |
| Gender Conformation and Associated Duties | “So then it turned into sexual abuse where he demanded that I preform my wifely duties, which was whatever he wanted and whenever he wanted… And if I did anything to step out of line, he would use it against me.” RW P1  “I was again, like, stunted in my adult growth so I was very much convinced that any failure in the relationship was my fault because, you know, he was the man of the house and if I just tried harder, it would be better.” RW P8  “…mum does what her husband or wife does what husband says to do and often have to ask if they can use the vehicle or go places. They are not usually feeling like they can just go and visit somebody, they have to stay home and do the housework. When I was asking someone to join in… programs, one of them said, my husband says I should stay home because our baby cries too much… ‘Your body is my body and we will have sex when I say so, it's not when you feel like it.’ … there's the financial part of it – lots of control over the money. It's my, ‘I decide what happens with our money.’” RW SP5D  “So partway, I would, in that relationship if I were not to completely baby them, or I would not give them money and be like hey you should, you need to go out and get a job and be able to provide for myself, right, they’d be like you’re treating me like a man, you’re invalidating my gender identity, you’re being manipulative and misogynistic” 2SLGBTQQ+ P17 | Both |
| Invalidating Gender Identity and Sexual Orientation | “She basically said that we're getting married, you're gonna have to not do not transition or anything else, because I'm marrying a man. And that's, that was like, well I kind of need to do this for myself to be in a better mental state. And she just kept me emotionally trying to bring it up. Every time we got a fight, she told me I was, like, every time we got into a little bit of an argument, she would bring up my transgender state, and everything else. So I had to basically live as my old self and everything else for her, for the most part, which was mentally exhausting for me.” 2SLGBTQQ+ P6  “But they said to me, it’s like oh well let me guess you have a boyfriend now or something, and I’m like again invalidating the fact, like it’s like you were never actually like bisexual, pansexual, you’re just like faking it for attention and I’m like okay, alright, and then the block button was a really good friend eventually and then they added me again and I was like holy crap and they were so sorry and I was stupid and I forgave them and then they started being a bit more like manipulative and then eventually, it, this is completely unrelated but I posted a picture of. I went out to dinner with my brother who they-they knew, or I went out to lunch with my brother and they knew like, like I have brothers, right? And they got so mad they were like who you going out to dinner with, blah blah blah, and I’m like hey were not together but anyway this is my brother and then they got embarrassed and they unadded me, so, ya, it was just constant attacks and like suspicion, and trying to prove that I was never actually, like part of the LGBTQ community, and guilting…” 2SLGBTQQ+ P17 | 2SLGBTQQ+ |
| Threatening to Out Victims | “A lot of the times when she wasn't happy with what I did, she would threaten by saying that she would out me to my parents.” 2SLGBTQQ+ P16  “We see a lot of control as well as some things unique to the 2SLGBTQ+ folks around being more isolated, outing to family, some of those sorts of issues that are unique to that particular client group.” 2SLGBTQQ+ SP2  “I think a lot of it is shame. A lot of it is, we hear a lot of one partner threatening to “out” another partner. That's a big thing that is kind of common to that community and does constitute abuse and some people don't always see it that way.” 2SLGBTQQ+ SP4  “Dictating if and when they can share those experiences with loved ones or people within their network and so um, essentially like, deciding if and when somebody is able to out themselves or if they then take away that power and decide to out them, as a way to sort-of exercise that control or coercion.” 2SLGBTQQ+ SP5 | 2SLGBTQQ+ |
| Unfaithful | “Yeah, because I wasn’t allowed to before or I’d be accused of doing you know, like, shady things the few times i’d, you know, go into town for a show or something. I used to be a musician so that was normal when we used to live in the city and he’d be like ‘You’re doing shady shit’, you know, and I’d have to send him pictures of the fingers, right?” RW P8  “[The participant and their friends] went to go visit [the friend’s] family and their grandmas and stuff, and we 've were gone over the weekend. And we came back and my-my partner at the time was very, like, I was always just consistently being like, ‘Oh, you're just gonna go on this trip and be a hoe, you're just gonna go on this trip and be a flightslut, you're nothing betterbut a more mattress ornament.” RW P11 | Rural Women |
| **Theme 2: Emotional and Sexual Manipulation** | | |
| Gaslighting | “He kept telling me people were telling him all these things that were said. I wasn’t telling anybody, like, so, then he just kept trying to gaslight me and I don’t even know. It was just a bunch of mental mind games.” RW P1  “We were getting together for birthdays; he was coming over to come pick up my daughter to take her on a birthday, like date, right? There was something that I didn’t agree with, I didn’t like the way something was being handled and I asked if we could talk about it, so I pulled him into the other room and it was the same old same behavior that – it was a me-thing, that I’m the problem here, look I just need to do his thing, he’s wanting to take my daughter out, I should feel lucky, all of this stuff. So, I said, ‘No, no, I think you need to leave.’ I said, ‘I’m not comfortable with this at all.’” RW P1  “So the dynamic of my ex-husband also comes into this. He has remained as a support system to me, and seems somewhat empathetic as to what I’ve been through, yet at the same time would like to shame me a little bit about ‘I told you so’, as he still, I think still trying to figure out...” RW P10  “Things like why can't you just take a joke, you're so easily, you know, you're so easily like, you get upset, so easy and constantly it was like, oh, yeah, I am right?. I'm a just a sensitive person, right?” RW P11  “He was clingy to a point where if I did anything that wasn't centered around him, he would get so upset. And you know, it would always be like, you know, ‘why are we paying attention to me? Why aren't you giving me attention or whatever?... How do question me? Don't Love me? You know, how could you as someone else's opinion influenced you?’” 2SLGBTQQ+ P2  “He did try to trick me into having a threesome, even though I had repeatedly expressed that I wasn't attracted to him. And he lied to me about what my partner wanted. This means, yeah, to convince me to come into the room and start something Most of what he did was just very sneaky, underhanded manipulation.” 2SLGBTQQ+ P3  “He basically told me that I blamed everything on my mental health said that I was crazy, said that I was bipolar and that I was making everything up in my head. He cheated on me with, I think, five other people and ya.” 2SLGBTQQ+ P9  “There was a lot of, like, cheating and then blaming me for it.” 2SLGBTQQ+ P14  “I think one of the things that I do remember that was a big thing was like a lot of emotional manipulation or gaslighting, I think towards like the end of it, I would say that I was moving out, and then well, actually, she would tell me that I need to move out. And then I would tell her that I was moving out, I was looking at different places. And then she would go back on her words and say, No, I don't know why you're moving out, because you signed a lease with me.” 2SLGBTQQ+ P16  “I was like, receiving help, and I was in a support group. It made it a lot easier for that partner to like villainize me in like a sense where sometimes I would just get like so overwhelmed or I would just like really want to be alone to just like cry and kind of like break down because I was still like digesting everything that had like happened like with the previous partner and then on top of that losing my job and then losing my apartment, and so, because I was in such an imbalanced like mental state like he would always like he would always like he was basically would like tell people that he was like scared of me and that I was like because I was like always like always having these mood swings and stuff I was like unstable and he would just like make me feel really bad to the point where I just wanted to just be by myself. I do have like mental health issues and I need to like work on and things like that but like what I didn't realize was like that. He was like prying on that and like was like pushing it like, out of like my window tolerance and that's why I would have like meltdowns and shut down and things like that, he was making me feel guilty that he was basically like, pushed me to a point where I was like, overwhelmed and was like, acting out. And then he would like blame me for getting overwhelmed and so it was like really confusing.” 2SLGBTQQ+ P18  “So it undermines their authentic experience by them saying ‘no, I am part of this community.’ And they say, ‘Oh, no you’re not, you just need someone to pay attention to you.” 2SLGBTQQ+ SP1  “An assault happened to him by his partner while he was in the shower, because the other partner saw that he had a drink with him in the shower and he got quite upset and agitated because ‘you can’t even have a shower without having a drink.’” 2SLGBTQQ+ SP1  “Often perpetrators of violence or abuse will use somebody's mental health to further perpetrate more abuse… [it’s hard to] realize that, like, they're not crazy and that they do have, like, they do have the mental health resources or capabilities to make change or do the things that they want in their life without having to rely on the perpetrator.” 2SLGBTQQ+ SP2  “I think that a lot of times they don’t see it as an IPV. I think that it’s manipulated, especially – so if we look at disordered eating. What we see is that it’s looked at as a concern versus an IPV. So ‘they’re concerned for my wellbeing, so they want this’ or ‘they’re trying to help me in getting better.’ But again, there’s all the shame, the abuse, the emotional abuse, the guilt, that’s placed on them for their different physical manifestations, right.” 2SLGBTQQ+ SP7 | Both |
| Shaming Financial Independence or a Lack Thereof | “This individual shamed and guilted me for choosing to live alone and support myself and pay my bills instead of living with them. According to them it was a way that we could save bills instead of pay 2 months of rent basically where they were staying, where I was staying. It was a way to combine our expenses and pay one rent. And so, I was shamed and guilted for basically choosing to live alone or having my own independence.” 2SLGBTQQ+ P8 | 2SLGBTQQ+ |
| Nobody Else Will Want You/Not Good Enough | “So now they're holding, your partner can hold that over you so easily like, you know, you can't hold down a job, no one’s going to help you, you’re halfway through this process, who’s gonna take care of you, and they can hold that over their heads… When you don't know who to trust or who's gonna accept you and your partner is telling you that nobody accepts you for who you are, only them, it’s hard to hold down a job…when somebody's been telling you that you're nothing, you have nothing, nobody will do anything for you and you're not worthy, that, you know, the fear of, knowing OK well at least I have this person and, you know, I, you know, I can kind of see when they're escalating and I can kind of determine these things, it’s known to me.” 2SLGBTQQ+ SP4  “A partner telling their significant other that, you know, ‘ever since you started taking tea, you’re not as attractive… any pattern of behavior that accumulates overtime, it is repetitive, that ultimately ends up affecting someone self-esteem or their self-image and so – something examples that come with coercive control is someone saying ‘why are you wearing that dress I don't like that dress on you’ or for instance someone making a comment such as ‘I actually don't think that person is a good friend, you should really think about that’ already just implants those notions of distrusting our own autonomy and, and ourselves, and so I think when we talk about coercive control, that can really play into people preventing – preventing people from coming forward.” 2SLGBTQQ+ SP8  “Thinking about, you know, for instance, is the partner a prominent member of the community that they are afraid of what other people might think or say, maybe depending on the status of affluence that person has, but even if they aren't part of the 2SLGBTQ+ community, you know wondering what others might have to say about that, or not wanting to believe that, you know, what they are experiencing is intimate partner violence because of, because of their own ideas and understanding of what intimate partner violence looks.” 2SLGBTQQ+ SP8  “A lot of that mental health piece. You know, because they’ve been told they’re not good enough, they don’t know how to do things, they don’t feel that, they can even, like what’s the point of even asking for help. I don’t know how to do it myself anyway, or that feeling of, like well I, you know, because they’ve been told they [don’t] deserve better or what have you that they just don’t feel like they should tell anybody. There’s feelings of like guilt and shame or tightly wrapped around that or, you know, I think it’s a lot of psychological things that are involved.” RW SP8 | Both |
| Emotional Manipulation to take Sexual Advantage of the Victim | “When he’d get really mad, and I’d get all apologetic, you know, what can I do? What can I do? He would use that as, you know, an excuse to, you know, not force, but I’d be guilted into doing lots of things.” RW P8  “Like, it started being, he would have tantrums about like sexual stuff, if he didn’t get sex or he didn’t get oral sex, he would start having tantrums... first it was tantrums. It was more coercive I would say. Like, it was easier to just get it over with rather than him throwing a fit for three days or like turning off the oven in the middle that I’m cooking.” RW P9  “[The third individual] never physically hurt me because he wasn't that stupid. But like, he knew that I had been through a considerable amount of abuse, I was still being abused by my parents when he was in the picture. So he knew that I knew how to recognize abuse a mile away. So he never tried anything super obvious. But this one time, he did try to trick me into having a threesome, even though I had repeatedly expressed that I wasn't attracted to him. And he lied to me about what my partner wanted.” 2SLGBTQQ+ P3  “there’s more, some sexual stuff, like me just giving in cause they won’t stop. And that happened on quite a few occasions but I remember one time, they wanted to, like, use toys on me, and I really didn’t sort of want to, and I said no, not sort of didn’t want to, I didn’t want to and I said no. But we’re still playing with the toys on him, and then, back on me again, and kept asking me and then he just put it right in. So he was like sort of toying with it on my hole, and then, again I had said and then he just jammed it in, it was lubed and everything but you know.” 2SLGBTQQ+ P15 | Both |
| **Theme 3: Threats of Death by Suicide** | | |
| N/A | “It didn’t really clue in until the day like, like he kept coming over to my house and dropping stuff off, and saying he was going to go kill himself, and stuff.” RW P3  “[The partner] actually used the suicide from my son… And then he told me, just all the ways he was going to [engage in death by suicide]… he said it would involve people, and I’m like… you can’t place this on me cause you knew my son committed suicide. So, he knew where to get me with certain things.” RW P3  “He did once hold a gun to his own head and threaten to kill himself, which I suppose swayed my decision to seek help because then I was convinced I was the perpetrator and I was doing this to him.” RW P8  "The thought of having someone die because of you, of your choices, that's absolutely overwhelming.” RW SP5  “They’re doing it to control the individual, right, to make them scared, to make them feel like you know if I leave you I’m gonna kill myself so that they don’t leave. It’s part of that power and control wheel for sure.” RW SP8  “She also suicide baited me a few times.” 2SLGBTQQ+ P3  “So, after she made, of what I thought was idle-suicidal threats, she tried to hang herself. I heard the sounds and knew that that was truly what she was trying to do. I told her that if she couldn’t open the door I was going to call the cops for a wellness check because I had already stated that I was going to leave, and with this happening I knew 100% that that was what I was going to do, because I’m not going to be responsible for her choices in that moment, that’s something I’m not equipped to handle, I would just call the cops and let them do a wellness check with her. She very quickly opened the door with strangulation marks around her neck and scratches on her chest and she told me how could I leave her at a time like this, it wasn’t fair and I didn’t know what I was doing and how detrimental it was that I leave at this particular point in her struggle... She had caused me to lose my job, she came into my job threatening suicide right at my job, I was a barber and she came into the shop and she was like hey if you don’t come home right now and you don’t stop doing what you’re doing, I am just going to like end my life right now, right here.” 2SLGBTQQ+ P8  “And it was really hard to leave him, every time I tried to leave him he would threaten suicide or he would aggressively come after me.” 2SLGBTQQ+ P9  “Using suicide, and suicide attempts… Ya, and then there’s incidences of suicide. Can’t leave me, I’ll kill myself. I wouldn’t be able to live without you. And actually going to the high level bridge. Like a text I’m at the high level bridge now, goodbye.” 2SLGBTQQ+ P15  “Financially, I was fine, they would always ask me for like rent money or whatever or they would like kill themselves... Emotional would be if you don’t come over I’m going to kill myself, stuff like that, and just playing like those mind games... They had reached out in like May or something and they kind of like tried to play like a manipulative like game with my head. They’re like oh, "ya I was so sad when you left I jumped off a bridge”. That never happened by the way I talked to their brother’s girlfriend and she’s like that never ever happened and even the brother was like that never happened.” 2SLGBTQQ+ P17 | Both |
| **Theme 4: Apologies and Vacuous Promises as Components of the Cycle of Abuse** | | |
| Apologetic with Kindness and Promises that Change will Occur (Cycle of Abuse) | “It was constant communication trying to get me to come back. A lot of the empty promises and manipulation, etc..” RW P1  “Like I mean, like he asked me, what could he do to get me, for me to come back to him. And he said to me, I will do counseling. You know, and so I, he said I don’t know where a counselor is. So I phoned my, I was talking to my counselor so she said, gave me some names in the [name of area]… for him to come. He said well I will go if you pay for it. I’m going, I’m having a hard time. So he was trying to drain my-my-my account. I didn’t have much money to start with because he kept draining it anyways. So.” RW P2  “And then he comes by with the gaslighting and the love-bombing and apologies and all that stuff, and I was still in love with him and all that, you know.” RW P9  “But I wonder what it would have taken for me to know that I was being abused. I just was so loyal to what he told me: ‘No, there’s nothing wrong with you’; ‘No, I love you’; ‘No, I would never hurt you’. So, as the confusion started to drip into my life, I don’t know. I don’t know what, I don’t know how I would have helped myself. “The silent treatment started, the interjection of massive gifting and attention and followed me to xxxxxxxx when my grandma was dying to be there as a support system.” RW P10  “It’s very like Latino in some ways that you just found and together; you ride or die together and, and so, and he really used that opportunity to kind of be like, hey, I can help you with [name omitted] and I was like, wow, that noble guy and like the wow, he-he's really changed. And so then I actually ended up going to the courthouse and saying I lied about that whole incident.” RW P11  “Like, 2 or 3 weeks after the restraining order expired, she actually sent me an email and it was a huge email and she was telling me all about her life and she’s asking if we could be in contact again. If she could see my child and she feels like she would really benefit from it and she would, you know, grow to appreciate my ex in her life again, and, basically just a huge bunch of bullshit. So (laughter), so I just, she’s like if you ignore this or you don’t answer this I’ll totally take it as you don’t want that but like I’d really like to have that. I feel like we’ve both really grown, we’ve both really matured and we’ve both learned from our experiences and I was just like are you kidding me?” 2SLGBTQQ+ P8 | Both |
| Trauma-Bonding/Needing the Victim | “They seem nice, and then after it’s like they kinda get ya feeling sorry for them kinda thing?” RW P3  “I’ll never forget this because, even though, it sounds strange that my husband would have taken [me to? 21:29] the hospital, he was still complaining how was he going to manage without me. I was, like, practically near death at that point in all respects.” RW P4  “He would I mean, he'd come and knock at the door and be like, please, I just want to see you, I just need to see you.” RW P11  “Something that we noticed as an ongoing trend is that one of the partners would struggle with mental health and the other one was trying to get through it but sometimes that escalated into either frustration on the part of the person who did not struggle with mental health or you know, maybe, not the best reactions or the most relationship-strengthening reactions by the part of the person who did struggle with mental health.” 2SLGBTQQ+ SP1 | Both |
| **Theme 5: Using One’s Parenting and Children’s Wellbeing to Manipulate Partners** | | |
| The Parental Sphere | “He was expecting me to parent my children the way he wanted me to parent them. So, my relationship with my two kids became extremely strained because I had to perform the way he wanted me to. Or there was some kind of threat, right. I was scared, so I very much did as he told me to, to try and salvage the relationship, or in the moment what I thought was to better myself. I thought it was a problem with me, so I thought I was growing and learning, but in hindsight I can see that what he was asking and telling me to do was an extremely unhealthy parenting attitude… So, I, yeah, I started cutting myself. I would go in the bathroom, sit in the tub, and cut my legs. He started telling me that – because he would eventually see the cuts on my legs – he’d tell me that if I kept cutting, that he would report me to, I don’t know, Alberta Mental Health, tell them that I was an (can’t hear) mother. So, because he had his phone in his hand and was going to call them, I promised I wouldn’t do it anymore. But privately I did do it.” RW P1  “I mean that is the thing, I mean they use kids as a leave reach. Like, I mean I was told many-many times that I couldn’t, if I left, I wouldn’t be able to have the kids. And so I thought, there’s no way I’m leaving my kids with you, you know, at least I know what’s going on, even though maybe I might not have been the best help that they had at the time, because you were just surviving yourself not realizing.” RW P2  “[The abusive partner says that they] miss my kids.” RW P11  “And, yeah, and the children, right, I think that's one of the biggest challenges I find that clients face when, when yeah, with IPV, because, you know, there's one, if a client chooses to leave their situation, they often worry about access to their children, you know, they're often threatened if you choose to leave, then you won't see your children.” RW SP1  “Want to keep their family together… they don’t love the behavior, but they love their spouses… their ability to blame themselves is unbelievable, and their partner reinforces it for them regularly.” RW SP2  “’You’re not smart enough to live on your own’, or ‘I’m going to call CFS and have the children taken from you, you’re a bad parent, you don’t know how to live on your own.’” RW SP8  “People who perhaps have been common law and had the children together through whichever ways they do that, but the gestational parent, if there is a gestational parent, yeah, at least threatens to take away rights of the other parent or, sort of, holds the kids over the other person's head.” 2SLGBTQQ+ SP2  “Who’s abusing you is the biological child's parent, they can hold that over you too and be like ‘you have no access to the child if you leave me. I don't care that you’ve raised them for how long. You’ll have to go to court. But, you know, I can still fight you and you'll never see them again.’” 2SLGBTQQ+ SP4 | Rural Women |
| **Theme 6: The Use of Health Conditions and Faking Illness** | | |
| Shaming the Use of Medical Care | “So controlling access to medication, controlling access to medical care. So you know, making people feel shameful about some of the medical care that they need in order to be themselves as well as coercive control.” 2SLGBTQQ+ SP3 | 2SLGBTQQ+ |
| Shame Related to Transitioning | “Again, we are starting to see individuals that are transitioning and we’re hearing more about how the medication is affecting their ability to eat, but then again with IPV and we know with anxiety and depression it’s sometimes a struggle to eat, or you know, so there’s those disordered eating. But also with obesity, sometimes with the gaining of the weight, and then the shame that the perpetrator is putting on them around that, yeah.” 2SLGBTQQ+ SP7 | 2SLGBTQQ+ |
| Faking Medical Illness | “He actually faked a disability so he stopped working shortly after we got married too and put a huge financial burden on me. So, I had to work fifty or sixty hours a week to make ends meet because he just up and quit working when he had, I would say, a high paying job before. He was in the oil and gas industry. So, I didn’t know at the time obviously that he faked the disability, but then he would sit at home for like twelve or fourteen hours a day playing video games but if I asked him to do a load of laundry or like a load of dishes, it was the end of the world. It was a four-day fight… He would guilt trip me because he’d be like ‘I’m disabled and you’re abusing me’, like ‘You’re an abusive caretaker’, that kind of thing.” RW P9  “Emotionally manipulated me into believing that she had stomach cancer. So that was a whole play on my caring nature and the part of me that really likes taking care of people and really affected how well I took care even of people in my family. So with that one I lost my job, I lost my home, I lost everything at the end of that relationship.” 2SLGBTQQ+ P7  “I found out later they lied to me about being re-diagnosed with pancreatic cancer for the second time. I didn’t know that she had lied about this until we broke up and she told me about 9 weeks after being with her that she had started chemo and radiation and it was on a weekly basis. There were numerous excuses and reasons why I was never able to, be allowed to join the appointments or join when results were given or join when any procedures were administered. I was blamed for cancer, suicidal thoughts and ideations that were involved with supposedly these treatments that she was having and for any other reasons why she may feel suicidal, why she couldn’t work. I was- it was somehow put on me that I-I was to blame... When I asked her to go and get a job to help financially, she chose instead to overdose on medication that I had in the home and then she blamed me because of what I asked her to do and said that she basically felt like I was telling her that she wasn’t good enough because she was not working. After stating that mental health help was required for her regularly and suggesting that she do that to maintain some regularity and some stability in her life, she shut me out of her suicidal thoughts and instead blamed me for the self-harm that ensued afterwards... she was, according to her, going through cancer and she had finished her chemo and radiation treatments and she was trying to recover, she felt like she’d rather like to be closer to family so she manipulated me into feeling like that that was the only choice I had to make her happy.” 2SLGBTQQ+ P8 | Both |
| Use of COVID-19 | “And so one of the things is that COVID-19 has been absolutely weaponized by abusers in several facets. So one, it's like, you can't go to that hospital, because if you do, you're gonna get COVID. And so that was kind of the most common thing that we saw, right off the hop when pandemic first started.” RW SP5 | Rural Women |
| **Theme 7: The Use of Religion or Faith to Reinforce Gender Standards** | | |
| N/A | **“**Lots of spiritual abuse. We both claimed Christianity, so there was lots of twisting of scripture and guilt, shame, and condemnation, used with that.” RW P1  “The Church worked with us [and was affiliated with my abusive partner] in trying to get us into counselling, but they had to choose the counsellor, so they chose an egotistical male that [name]completely brainwashed (big sigh). Anyway, so, there was continuous manipulation through the church, through the pastor, through the counsellor…” RW P1  “He got me involved in a church that, and just basically God held me to all these standards, and I wasn’t religious at the time.” 2SLGBTQQ+ P9 | Both |
